# Comparative *In Vitro* Study of Biofilm Formation and Antimicrobial Susceptibility in Gram-Negative Bacilli Isolated from Prosthetic Joint Infections

**DOI:** 10.1128/spectrum.00851-22

**Published:** 2022-07-25

**Authors:** Alicia Macias-Valcayo, John-Jairo Aguilera-Correa, Antonio Broncano, Raul Parron, Alvaro Auñon, Joaquin Garcia-Cañete, Antonio Blanco, Jaime Esteban

**Affiliations:** a Department of Clinical Microbiology, Instituto de Investigación Sanitaria (IIS)-Fundación Jiménez Díaz, Universidad Autónoma de Madrid (UAM), Madrid, Spain; b Centre for Biomedical Research Network in Infectious Diseases (CIBERINFEC), CIBER de Enfermedades Infecciosas, Madrid, Spain; c Department of Orthopaedic Surgery, Fundación Jiménez Díaz, Madrid, Spain; d Department of Internal Medicine-Emergencies, Fundación Jiménez Díaz, Madrid, Spain; Hartford Hospital

**Keywords:** prosthetic joint infection, biofilm, Gram-negative bacilli, Gram-negative bacteria

## Abstract

Prosthetic joint infections (PJIs) are typically caused by microorganisms that grow in biofilms. Traditional antimicrobial susceptibility tests are based on the study of planktonic bacteria that might lead to missing the biofilm behavior and to a treatment failure. This study was designed to analyze the antimicrobial susceptibility of clinical Gram-negative bacilli (GNB) isolates from PJIs in planktonic and sessile states and the possible relationship between antimicrobial resistance and biofilm formation. A total of 46 clinical isolates from patients with PJIs (mainly hip and knee prostheses) plus three GNB ATCC isolates were studied. The Minimal Inhibitory Concentration (MIC), minimal bactericidal concentration (MBC), minimal biofilm inhibitory concentration (MBIC), and minimal biofilm eradication concentration (MBEC) were assessed using a previously published methodology. Almost all of the GNB clinical isolates tested were biofilm forming. Pseudomonas aeruginosa was the largest biofilm-forming species. A comparison of MBIC_90_ versus MIC_90_ shows an increase higher than 1- to -2-fold dilutions in most antimicrobials studied, and MBEC90 was significantly higher than MIC90, becoming resistant to all the antimicrobial drugs tested. Higher biofilm production values were obtained in antibiotic-susceptible Escherichia coli in comparison to their resistant counterparts. However, regarding the relationships between antimicrobial resistance and biofilm formation, our analysis showed that each strain differed. A high antimicrobial resistance rate was found among the GNB studied. Moreover, almost all bacterial isolates were *in vitro* biofilm formers. Although there was no significant association between biofilm and antibiotic resistance, multidrug-resistant isolates were found to be greater biofilm formers than non-multidrug-resistant isolates.

**IMPORTANCE** This study is the first one to analyze a high number of isolates of Gram-negative bacilli that are the cause of prosthetic joint infection. The analysis includes biofilm development and antimicrobial susceptibility testing of both planktonic and sessile bacteria. The obtained results support the clinical knowledge about the treatment of these bacteria when biofilms are involved.

## INTRODUCTION

Prosthetic joint infections (PJIs) are an important complication in orthopedic surgery and are often associated with additional clinical procedures, prolonged hospitalization, higher health care costs, and mortality (1, 2). Infection may be acquired around the time of surgery or via hematogenous spreading from a distant foci; rarely, they may be caused by spread from contiguous foci (3). Overall, the most frequent microorganisms causing PJIs are coagulase-negative staphylococci, followed by Staphylococcus aureus, Streptococcus spp., Gram-negative bacilli, Enterococcus spp., and anaerobes (3, 4). Despite appearing as a relatively uncommon cause of PJIs, Gram-negative bacilli (GNB) appear to be increasing in proportion over the last few years (5). In a large series of PJIs in Spain over 10 years, Benito et al. reported a statistically significant rising linear trend for PJIs caused by aerobic Gram-negative bacilli (25% in 2003 to 2004 to 33.3% in 2011 to 2012), while aerobic Gram-positive cocci decreased from 80.3% in 2003 to 2004 to 74.3% in 2011 to 2012 (5). It is important to consider that selecting the most appropriate antibiotic therapy critically influences the outcome of the patient (6), and some of these infections are caused by multidrug-resistant strains of Gram-negative bacilli that are extremely difficult to treat (7). Moreover, patients with PJIs caused by Gram-negative infections tend to be older and show worse outcomes than those patients with Gram-positive infection, despite receiving adequate treatment (8). Another difficulty added to these complications is the formation of a biofilm on the surface of the implant that plays a significant role in the pathogenesis of PJIs (9). The first biofilm definition was developed by Costerton and co-workers, when they observed that bacteria stick on surfaces forming matrix-enclosed surface-associated communities (10). According to some experts, biofilm is the most successful life-form on earth (11). It consists of a cluster of microorganisms embedded in different types of biopolymers named extracellular polymeric substances (EPSs). This form of life provides diverse properties highlighting cell-cell communication, exchange of genetic information, a nutrient source, and a protective barrier. This barrier includes protection against desiccation and host defenses, as well as conferring tolerance to various antimicrobial agents (12, 13). In addition, in the final stages of biofilm development, several mechanisms are used by bacteria to disperse themselves, allowing the colonization of new surfaces and enhancing the dissemination of the infection (14).

Usually, antimicrobial susceptibility testing is performed using bacteria in the planktonic state, missing the biofilm characteristics that may lead to a treatment failure (15). The determination of MIC is the most common method for testing the ability of a compound to inhibit microbial replication, and it is useful in the treatment of many acute infections (16). However, therapies based on MIC in the treatment of chronic or device-related infections in which biofilm plays an important role may be ineffective (17), and surgical removal of the infected tissue or implant is mandatory in order to reach a good outcome for the patient. This study aimed to evaluate the biofilm formation capacity of 46 GNB strains isolated from PJIs, as well as the properties of these biofilms.

## RESULTS

Species were isolated from samples of different PJIs, including hip (50%), knee (41.4%), shoulder (4.3%), elbow (2.1%), and spinal (2.2%) infection. The median age of the patients was 74 years (interquartile range [IQR], 68 to 84). Around half of the patients (24 of 46) had had a prior prosthesis-related hospitalization. There were 32 acute infections (3 were classified as hematogenous), and 14 episodes were chronic infections. The most preferred treatment for acute GNB PJIs (25/32) was a debridement, antibiotics, and irrigation with implant retention (DAIR) procedure. In only one patient with chronic infection, DAIR was the surgical procedure chosen, although a surgery to remove and replace the prosthesis had been previously performed. On the other hand, prosthesis extraction and replacement were chosen in 16 patients (7 with acute infection and 9 with chronic infection). Ultimately, 2 patients were managed with implant retention, and there were 2 cases that required amputation (Table 1).

**TABLE 1:** Characteristics of 46 patients with PJIs^*a*^

Characteristic	Total (*n* = 46)	%
Age (years)		
Median	74	
Range	20 to 91	
Gender		
Male	20	43.5
Female	26	56.5
Localization		
Hip	23	50
Knee	19	41.4
Shoulder	2	4.3
Other	2	4.3
Polymicrobial infection	14	30
Acute	32	70
Chronic	14	30
Surgical strategy		
DAIR	26	56.5
One-stage exchange	3	6.5
Two-stage exchange	13	28.4
Antibiotic suppressive therapy	2	4.3
Amputation	2	4.3

a DAIR, debridement, antibiotics, and implant retention; PJI, prosthetic joint infection.

Regarding the microbiologic findings of the 46 episodes of GNB PJIs, there were 14 polymicrobial infections (30%): 7 were due to more than one GNB, and 7 were due to mixed infections that included Gram-positive cocci (3 Enterococcus faecalis, 2 Staphylococcus epidermidis, and 1 Cutibacterium acnes). The most frequently isolated organisms were Escherichia coli (8 patients) and Proteus mirabilis (8 patients), right after that Pseudomonas aeruginosa (7 patients) and Klebsiella pneumoniae (7 patients).

### Assessment of biofilm production *in vitro*.

The average optical density (OD) values showed a broad distribution between the different strains (Tables 2 and 3). According to this classification, most of the strains studied were biofilm producers (97%). From 46 bacterial isolates tested for biofilm formation, 19 (41.3%) were categorized as strong producers, 16 (34.8%) were categorized as moderate producers, 10 (21.8%) were categorized as weak producers, and 1 (2.1%) was categorized as a nonbiofilm producer.

**TABLE 2:** Comparison between EB and NFGBN biofilm formation^*a*^

Family group	No. of strains			Total
	Biofilm formation (Q1 to Q3) (*n*-fold OD_c_)	Nonbiofilm producer (%)	Biofilm producer (%)	
EB	3.28 (1.72 to 5.68)	1 (2.1%)	37 (97.9%)	38
NFGNB	10.06 (5.60 to 22.78)	0	8 (100%)	8

a EB, Enterobacteriaceae; NFGBN, nonfermenting Gram-negative bacilli; OD_c_, cutoff value three standard deviations (SD) above the mean optical density; Q, quartile.

**TABLE 3:** GNB biofilm formation^*a*^

Strain (*n*)	Biofilm formation (Q1 to Q3) (*n*-fold OD_c_)	Percentage biofilm producer (%)			
		Strong	Moderate	Weak	No producer
A. baumanii (1)	2.5 (1.9 to 4)	0	1 (100%)	0	0
C. freundii (1)	3.4 (1.9 to 3.9)	0	1 (100%)	0	0
C. koseri (1)	1.3 (0.7 to 1.6)	0	0	1 (100%)	0
E. cloacae (2)	7.2 (5.6 to 8.4)	2 (100%)	0	0	0
E. hormaechei (2)	1.7 (0.7 to 3.6)	0	1 (50%)	1 (50%)	0
E. coli (8)	2.1 (1.2 to 3.3)	1 (12.5%)	4 (50%)	3 (37.5%)	0
K. pneumoniae (7)	4.9 (2.3 to 7.4)	4 (71.4%)	2 (14.3%)	1 (14.3)	0
M. morganii (3)	5.9 (2.1 to 13.45)	2 (66.7%)	0	1 (33.3%)	0
P. mirabilis (8)	3.3 (2.2 to 5)	2 (25%)	5 (62.5%)	1 (12.5%)	0
P. vulgaris (1)	1.3 (1.1 to 2.4)	0	0	1 (100%)	0
P. stuartii (1)	2.9 (2.3 to 4.6)	0	1 (100%)	0	0
P. aeruginosa (7)	10.2 (6.4 to 19.6)	7 (100%)	0	0	0
R. ornithinolytica (1)	0.9 (0.7 to 1.3)	0	0	0	1 (100%)
S. marcescens (3)	2.6 (1.7 to 5)	1 (33.3%)	1 (33.3%)	1 (33.3%)	0
Total	3.6 (1.8 to 6.8)	19 (41.3%)	16 (34.8%)	10 (21.8%)	1 (2.1%)

a GNB, Gram-negative bacilli; OD_c_, cutoff value three standard deviations (SD) above the mean optical density; Q, quartile.

Among the tested species, P. aeruginosa (7 of 7), Enterobacter cloacae (2 of 2), Morganella morganii (2 of 3), K. pneumoniae (5 of 7), and P. mirabilis (5 of 8) stand out as strong biofilm producers. The most abundant strains were E. coli (8 cases), K. pneumoniae (7 cases), P. mirabilis (8 cases), and P. aeruginosa (7 cases), which were weak/moderate, moderate/strong, moderate, and strong biofilm producers, respectively.

Upon analysis of the biofilm formation of each species, no pattern was found, except in the case of E. cloacae, in which the isolates showed the same biofilm-forming capacity (*n* = 2, *P* = 0.6). Furthermore, no significant differences were found between each group of species and biofilm formation. However, only in the case of E. coli and M. morganii (*P* = 0.9) and P. mirabilis and S. marcescens (*P* = 0.1) was the biofilm formation similar.

### Antimicrobial susceptibility results of planktonic and sessile state.

Seven antibiotics were used to determine the MIC, MBC, MBIC, and MBEC values of the 46 clinical isolates. The overall percentages of resistance among all the Enterobacteriaceae (18) and nonfermenting Gram-negative bacilli (NFGNB) (8) are reported in Table 4. The obtained MICs showed that the Enterobacteriaceae isolates (18) were sensitive to meropenem (95%), followed by amikacin (87%), ceftazidime (67%), ceftriaxone (65%), gentamicin (55%), ciprofloxacin (53%), and colistin (50%). The high resistance to colistin is due to the intrinsic resistance to this antibiotic of certain bacteria that are included in this category.

**TABLE 4:** Planktonic and biofilm-growing bacteria antimicrobial activity among EB and NFGNB^*a*^

Antimicrobial	EB (18)								NFGNB (8)							
	MIC		MBC		MBIC		MBEC		MIC		MBC		MBIC		MBEC	
	S	R	S	R	S	R	S	R	S	R	S	R	S	R	S	R
AMK	33 (87%)	5 (13%)	21 (55%)	17 (45%)	30 (79%)	8 (21%)	0	38 (100%)	6 (75%)	2 (25%)	6 (75%)	2 (25%)	5 (63%)	3 (37%)	0	8 (100%)
CRO	25 (65%)	13 (35%)	19 (50%)	19 (50%)	20 (53%)	18 (47%)	0	38 (100%)	NT	NT	NT	NT	NT	NT	NT	NT
CAZ	26 (67%)	12 (33%)	21 (55%)	17 (45%)	22 (58%)	16 (42%)	0	100 (100%)	8 (100%)	0 (0%)	7 (88%)	1 (12%)	2 (25%)	6 (75%)	0	100 (100%)
CIP	20 (53%)	18 (47%)	17 (45%)	21 (55%)	17 (45%)	21 (55%)	0	100 (100%)	5 (63%)	3 (37%)	3 (37%)	5 (63%)	5 (63%)	3 (37%)	0	100 (100%)
CO	19 (50%)	19 (50%)	17 (45%)	21 (55%)	13 (35%)	25 (65%)	0 (0%)	100 (100%)	8 (100%)	0 (0%)	7 (88%)	1 (12%)	2 (25%)	6 (75%)	0 (0%)	100 (100%)
GE	21 (55%)	17 (45%)	9 (24%)	29 (76%)	20 (53%)	18 (47%)	0 (0%)	100 (100%)	6 (75%)	2 (25%)	4 (50%)	4 (50%)	4 (50%)	4 (50%)	0 (0%)	100 (100%)
MP	36 (95%)	2 (5%)	36 (95%)	2 (5%)	35 (92%)	3 (8%)	0 (0%)	100 (100%)	6 (75%)	2 (25%)	6 (75%)	2 (25%)	5 (63%)	3 (37%)	0 (0%)	100 (100%)

a The table shows planktonic (MIC and minimal bactericidal concentration [MBC]) and biofilm-growing bacteria (minimal biofilm inhibitory concentration [MBIC] and minimal biofilm eradication concentration [MBEC]) antimicrobial activity among Enterobacteriaceae (EB) and nonfermenting Gram-negative bacilli (NFGNB). AMK, amikacin; CRO, ceftriaxone; CAZ, ceftazidime; CIP, ciprofloxacin; GE, gentamicin; CO, colistin; MP, meropenem; NT, not tested; R, resistant; S, sensitive.

All NFGNB (8) showed susceptibility to colistin and ceftazidime, followed by amikacin, gentamicin, and meropenem each accounting for 75%, while ciprofloxacin accounts for 63%. Moreover, upon analysis of the antimicrobial resistance of each species, the comparison of MBIC_90_ versus MIC_90_ shows a more than 1- to 2-fold dilution increase in most antimicrobials studied. Additionally, we found that MBEC_90_ values were significantly higher than MIC_90_, becoming resistant to all the antimicrobial drugs tested. (Table 5).

**TABLE 5:** Comparison of planktonic and biofilm-growing bacteria antimicrobial activity^*a*^

Antibiotic	Species (*n*)	Range	MIC (mg/liter)		MBC (mg/liter)		MBIC (mg/liter)		MBEC (mg/liter)	
			p50	p90	p50	p90	p50	p90	p50	p90
AMK	E.cl (9)	0.125 to 128	4	8	16	32	4	16	>128	>128
	K.p (7)		4	16	8	16	8	32	>128	>128
	P.m (9)		4	8	16	32	4	8	>128	>128
	P.a (8)		4	4	4	16	4	32	>32	>32
CRO	E.cl (9)	0.03125 to 32	<0.03125	>32	0.125	>32	2	>32	>32	>32
	K.p (7)		>32	>32	>32	>32	>32	>32	>32	>32
	P.m (9)		<0.03125	<0.03125	<0.03125	<0.03125	<0.03125	<0.03125	>32	>32
	P.a (8)		NT	NT	NT	NT	NT	NT	NT	NT
CAZ	E.cl (9)	0.03125 to 32	0.0625	16	0.25	16	2	16	>32	>32
	K.p (7)		16	32	32	>32	8	>32	>32	>32
	P.m (9)		<0.03125	<0.03-125	0.0625	0.125	<0.03125	4	>32	>32
	P.a (8)		1	2	2	4	>32	>32	>32	>32
CIP	E.cl (9)	0.03125 to 32	<0.03125	>32	<0.03125	>32	0.5	>32	>32	>32
	K.p (7)		4	32	8	>32	8	>32	>32	>32
	P.m (9)		<0.03125	4	0.125	32	<0.03125	4	>32	>32
	P.a (8)		0.125	16	0.5	32	0.25	32	>32	>32
GE	E.cl (9)	0.125 to 128	4	4	16	32	4	16	>32	>32
	K.p (7)		>128	>128	>128	>128	>128	>128	>128	>128
	P.m (9)		2	8	16	64	2	4	>128	>128
	P.a (8)		2	>128	4	>128	4	>128	>128	>128
CO	E.cl (9)	0.125 to 128	<0.125	<0.125	<0.125	0.5	0.5	4	>128	>128
	K.p (7)		0.5	16	8	64	8	32	>32	>32
	P.m (9)		NT	NT	NT	NT	NT	NT	NT	NT
	P.a (8)		0.25	0.5	0.5	1	4	8	128	128
MP	E.cl (9)	0.03125 to 32	<0.03125	<0.03-125	<0.03125	0.25	<0.03125	0.5	>32	>32
	K.p (7)		0.0625	1	0.5	2	0.5	2	>32	>32
	P.m (9)		<0.03125	0.0625	0.125	0.5	<0.03125	0.0625	>32	>32
	P.a (8)		0.5	2	2	4	4	16	>32	>32

a The table compares planktonic (MIC and minimal bactericidal concentration [MBC]) and biofilm-growing bacteria (minimal biofilm inhibitory concentration [MBIC] and minimal biofilm eradication concentration [MBEC]) antimicrobial activity. E.cl, Escherichia coli; K.p, Klebsiella pneumoniae; P.m, Proteus mirabilis; P.a, Pseudomonas aeruginosa; AMK, amikacin; CRO, ceftriaxone; CAZ, ceftazidime; CIP, ciprofloxacin; GE, gentamicin; CO, colistin; MP, meropenem; NT, not tested.

### Relationship of antimicrobial resistance and biofilm formation.

**FIG 1:**
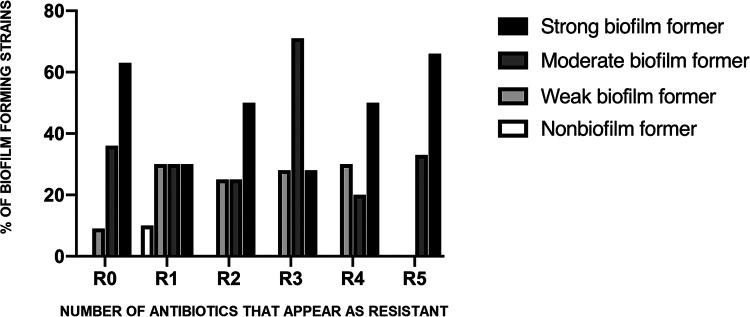
Relationship of antimicrobial resistance and biofilm formation of bacteria from prosthetic joint infections (PJIs). R0, no resistance; R1, resistance against one antimicrobial; R2, resistance against two antimicrobials; R3, resistance against three antimicrobials; R4, resistance against four antimicrobials; R5, resistance against five or more antimicrobials.

Regarding the relationships between antimicrobial resistance and biofilm formation, our analysis showed that they were different for each strain. Specifically, among nonbiofilm, weak, and moderate producers, no predominant pattern was observed. However, multidrug-resistant (MDR) and extensively drug-resistant (XDR) bacterial isolates (resistance to three or more antibiotic families and nonsusceptible to all but one or two agents, respectively) tended to be more biofilm forming than those isolates susceptible to all antibiotics studied or resistant to one or two antimicrobials (Fig. 1). However, the correlation between antimicrobial resistance and biofilm formation was not statistically significant.

Conversely, interesting data are observed in P. aeruginosa, in which the 70% of the isolates were susceptible to all the antibiotics studied though were the most biofilm forming isolates. In the present study, we also found a greater capacity to form biofilm in ciprofloxacin-susceptible E. coli, P. mirabilis, and P. aeruginosa isolates (Table 6 and 7).

**TABLE 6:** Global relationship between biofilm formation and antimicrobial resistance^*a*^

Antimicrobial	Biofilm formation (p50)				*P* value (GNB MIC/MBIC)
	MIC		MBIC		
	S	R	S	R	
Amikacin	3.3	7.42	3.3	7.42	0.0956/0.0619
Ceftriaxone	2.985	3.79	3.845	2.91	0.64/0.13
Ceftazidime	3	3.35	2.985	5.32	0.91/0.134
Ciprofloxacin	3.05	3.68	3	3.735	0.46/0.244
Colistin	3.61	2.97	3.11	3.79	0.68/0.054
Gentamicin	3.38	3.735	3.205	3.735	0.46/0.26
Meropenem	3.46	6.41	2.985	9.11	0.9/0.2443

a The table shows the global relationship between biofilm formation (p50) and antimicrobial resistance (MIC and minimal biofilm inhibitory concentration [MBIC]). GNB, Gram-negative bacilli; R, resistant; S, sensitive.

**TABLE 7:** Relationship between biofilm formation and antimicrobial resistance^*a*^

Antibiotic	E. coli (8)				K. pneumoniae (7)				P. mirabilis (8)				P. aeruginosa (7)			
	MIC (*n*)		p50	*P* value	MIC (*n*)		(p50)	*P* value	MIC (*n*)		p50	*P* value	MIC (*n*)		p50	*P* value
Amikacin	S	8	2.1		S	5	3.9	0.44	S	8	3.3		S	6	7.81	0.61
	R	0			R	2	3.36		R	0			R	1	18.06	
Ceftriaxone	S	4	3.055	0.56	S	2	3.74	0.44	S	7	2.97	0.51	S	NT	NT	
	R	4	2.228		R	5	6.3		R	1	3.37		R			
Ceftazidime	S	4	3.055	0.56	S	2	3.74	0.44	S	7	2.97	0.65	I	7	10.2	
	R	4	2.228		R	5	6.3		R	1	3.79		R	0		
Ciprofloxacin	S	4	3.055	0.56	S	0	4.9		S	4	4.38	0.24	S	5	19.2	0.2
	R	4	2.228		R	7			R	4	2.865		R	2	7.55	
Colistin	S	8	2.1		S	5	6.3	0.44	S	0	3.3		S	7	10.2	
	R	0			R	2	3.74		R	8			R	0		
Gentamicin	S	4	3.20	0.08	S	5	3.9	0.62	S	5	2.97	0.88	S	5	7.55	0.24
	R	4	2.16		R	2	5.8		R	3	3.79		R	2	19.2	
Meropenem	S	8	2.1		S	6	4.6	0.13	S	7	2.97	0.51	S	6	7.81	0.61
	R	0			R	1	10.28		R	1	3.79		R	1	18.06	

a The table shows the relationship between biofilm formation (p50) and antimicrobial resistance (MIC). R, resistant; S, sensitive.

## DISCUSSION

The implant of joint prostheses or arthroplasties is increasing rapidly worldwide. Although PJIs occur in 0.5 to 4% (9, 19–22) of arthroplasties, a PJI is a devastating complication, and the rate is likely to increase over the coming years. Moreover, the treatment of bone and joint infections caused by GNB is become more challenging. Possible explanations for the higher rate and the increased treatment difficulty include an aging population, comorbidities, and the implication of multidrug-resistant GNB. Here, this observation is consistent with our findings, since the median age was 74 years, and potential risks factors and high antibiotic resistance were observed.

Therefore, the data provided by our study could help physicians identify the microbiological spectrum, the tendencies of biofilm formation, and the antimicrobial pattern in GNB that would lead to treatment failure. During the study period from May 5, 2016, to August 9, 2018, the overall prevalence of GNB PJI rate was 23.83%. These results are comparable with other series conducted recently in Spain, where the percentage of cases of PJI caused by Enterobacteriaceae were 20.4% (5), in contrast with past series, in which GNBs were involved in less than 10% of cases of PJI (23). Moreover, our study conforms with those published in the literature that have reported higher frequency of these microorganisms in health care-associated infections (24–27). The most frequent species involved were E. coli, K. pneumoniae, P. mirabilis, and P. aeruginosa, similar to previously reported organism profiles (5, 8). There were no relevant trends for other isolated microorganisms, although the proportion of infections due to M. morganii (6.52%) Serratia spp. (6.52%), and Enterobacter spp. (8.70%) was greater than expected (5, 8, 28).

In this study, GN infections were more likely to involve to hip prostheses than other implants. Moreover, we observed a high number of polymicrobial infection (30%) especially in hip PJIs (57%) compared with knee (36%) and elbow PJIs (7%). As suggested by other authors, this fact may be due to the colonization of the hip and groin area with gut microbiota (29). Considering this, in our opinion, specific prophylaxis could be necessary depending on the joint where the prothesis is implanted.

A noteworthy finding in our study was the high biofilm producing ability of bacteria causing PJIs. Nearly all of GNB clinical isolates tested were biofilm forming (97%). Although there is no observed pattern at the species level, the tendency of these clinical isolates is to be producers of biofilm. Our findings differ from those of Gómez et al. (30) (in Spanish), who reported that only 36.25% of the Gram-negative bacilli studied by them were biofilm producers. Furthermore, their data showed that only 20% of the P. aeruginosa tested were strong biofilm producers, whereas in our study, all the clinical P. aeruginosa isolates were strong biofilm producers. These observed differences may possibly be explained by the origin of the strains. In this regard, it was recognized that the first step prior to any infection by a microorganism involves adaptation to the environment, leading to colonization and biofilm production as an important strategy for survival. Therefore, as Lebeaux et al. (18), Di Domenico et al. (31), and Kwiecińska-Piróg J et al. (32) suggested, the presence of implants in body sites probably facilitates biofilm formation, which is in concordance with our results. In our opinion, the characterization of biofilm formation by GNB clinical isolates from infections associated with prosthetic devices may help to identify those strains that show more of a tendency to form biofilm and consequently may affect clinical decisions and patient outcomes.

Regarding the evaluation of antibiotic susceptibility of GNB causing PJIs, the percentage of resistant strains was higher in comparison with other studies in the literature. Concerning resistance to specific antibiotics, such as ciprofloxacin because of its important role in treatment, our data show a notable nonsusceptibility percentage (47%) among the recovered Enterobacteriaceae (EB) in our study, whereas in other series, the proportion of resistance ranges from 0 to 19% (5, 28, 29, 33). In addition, 12 of 38 (33%) isolates of the EB were resistant to third-generation cephalosporins. The high antibiotic resistance rates observed among the GNB are a cause of concern because they greatly limit therapeutic options and are related to higher failure rates. It is important to consider that these clinical strains come from elderly patients. Some patients are recognized as reservoirs of resistant bacteria because of their frequent exposure to antibiotics and because of the higher rates of infection in hospital and institutional settings and therefore the high rates of antimicrobial prescriptions (34). The negative implications in the outcome of emerging antimicrobial resistance among the GNB from PJIs has also been published (5, 7).

According to previous studies, we expected to find a higher incidence of antibiotic resistance patterns by biofilm-producing bacteria (35–37), but there are several discrepancies among the different studies in the literature. In this sense, our results indicate high variability between biofilm formation and antibiotic resistance patterns among our isolates. Actually, although some isolates with higher levels of resistance tended to form stronger biofilms, the association was statistically insignificant. Moreover, we did not observe this association among all the collected isolates. For example, we found that ciprofloxacin-susceptible E. coli and P. mirabilis isolates tended to produce more biofilm formation than their resistant isolates. These results are consistent with previous studies on ciprofloxacin-susceptible K. pneumoniae (38), E. coli, and Salmonella Typhimurium (39) in which the acquisition of quinolone resistance has been related to a decrease in biofilm production. In addition, in agreement with the study led by Cepas et al. (39), we found that ciprofloxacin-susceptible P. aeruginosa isolates showed a greater capacity to form biofilm than the resistant isolates.

Thus, despite intensive investigations over the last few years, we are far from identifying the molecular mechanisms that involve tolerance of biofilm bacteria to environmental circumstances. This may be partially explained by various mechanisms that are responsible for this increased resistance, including restricted antimicrobial penetration into biofilms, decreased bacterial growth rate in the biofilms, the expression and exchange of resistance genes among bacteria, and the presence of inactivating enzymes in the biofilm (40–42).

Our research has several limitations. First, biofilm formation in our study was performed under *in vitro* conditions and may not precisely correlate with biofilms formed in medical implants *in vivo.* In fact, several factors have been reported that mediated biofilm formation, such as environmental conditions, host factors, and certain microbial isolates. On the other hand, our study was performed with a significant number of GB bacteria. Nevertheless, further studies with a larger panel of GN bacteria isolates and species from PJIs are necessary for confirming our findings. Moreover, another main limitation of the study is the relatively low number of strains of some species. Although we have included all available strains, the relatively low number of PJIs caused by Gram-negative bacteria prevent us from obtaining more strains in order to avoid this limitation, so the results obtained must consider this fact. Future research probably needs to include more strains (even using multicenter studies) for confirming our results.

In conclusion, knowledge about the relationship between the activity of antimicrobial drugs and biofilm production among GN bacteria is scarce. In fact, no standardization of biofilm antimicrobial susceptibility testing and breakpoints by official agencies have been published. However, the concentration of antibiotics necessary to eradicate the biofilm is many times higher than that for planktonic bacteria, with quinolones showing the best activity.

## MATERIALS AND METHODS

### Study design and data collection.

Forty-six strains isolated from patients with PJIs were included in the study. All of the strains were isolated from samples of patients with PJIs in the Clinical Microbiology Department of a metropolitan university hospital. All PJIs were diagnosed according to the internationally accepted criteria of the Infectious Diseases Society of America (IDSA) (43) Sociodemographic data (age and sex) and clinical data (history of repeated infections, surgery, and antimicrobial treatment) were collected. The study was approved by the institutional ethics committee (approval EO053-21_FJD).

### Isolation and identification of bacteria from clinical specimens.

Clinical specimens were collected in sterile containers and then transferred to a microbiology laboratory for immediate processing. The samples were processed by sonication according to the methodology previously described by our group (44). In summary, 50 mL of phosphate saline buffer was added, and the tubes were vortexed and sonicated in an Ultrasons-H 3000840 low-power bath sonicator (J.P. Selecta, Barcelona, Spain) at 22°C for 5 min, followed by an additional centrifugation. After centrifugation, the resulting pellet was cultured in different growth agar plates: tryptic soy agar with 5% sheep blood, chocolate agar, MacConkey agar, Schaedler agar with 5% sheep blood, and Sabouraud-chloramphenicol agar, all from bioMérieux (Marcy-l’Étoile, France) and incubated at 37°C and 5% CO_2_ for at least 24 h and up to 7 days, with the exception of Sabouraud-chloramphenicol agar, which was maintained at 30°C for 4 weeks. All of the microorganisms isolated were identified using matrix-assisted laser desorption ionization-time of flight (MALDI-TOF) (VitekMS, bioMérieux, Marcy-l’Étoile, France) and subsequently frozen and stored at –80°C.

### Biofilm quantification.

The biofilm formation was evaluated using a modified method of Stepanović et al. (45). Strains were defrosted in aseptic conditions onto tryptic soy agar with 5% sheep blood. To assess bacterial biofilm formation, a few colonies from an overnight culture plate of each strain were resuspended in a tryptic soy broth culture (BD, Germany) supplemented with 1% glucose to obtain a suspension of 0.5 on the McFarland scale (10^8^ colony-forming units/milliliter, CFU/mL). The obtained suspension was vortexed and thereafter diluted 1:100 in tryptic soy broth culture supplemented with 1% glucose. Each suspension was vortexed, and then 200 μL was poured into eight wells from a sterile flat-bottomed 96-well polystyrene microtiter plate (MicroWell, Thermo Scientific, USA). The experiment was performed in triplicate (for each strain, *n* = 24). A negative control was also included (200 μL of tryptic soy broth [TSB] supplemented with 1% glucose/well).

The plates were incubated aerobically under static conditions at 37°C for 24 h. After incubation, the supernatant from each well was carefully removed and were washed two times with 200 μL of sterile phosphate-buffered saline (PBS). After washing, the remaining adherent bacteria were fixed using 200 μL of methanol for 20 min and left to air dry. Once the microplate was air dried, we added safranin stain for 15 min for staining the biofilm. Prior to measurement of biofilm, the protocol includes resolubilization of the dye to measures the biofilm produced on both the bottom and the walls of the well. The last step was the measurement of results. The optical density (OD) of each well stained with safranin was measured at 492 nm by a microtiter plate reader.

Interpreting the results requires defining a cutoff value that separates biofilm-forming strains from non-biofilm-forming strains. In the present study, we chose the statistical test described by Stepanović et al. (45). The average OD values of the negative control and for each strain tested was calculated. Second, the cutoff value (OD_c_) was defined as three standard deviations (SD) above the mean OD of the negative control: OD_c_ = average OD of the negative control + 3× SD of negative control. The final OD value of each strain tested was expressed as average OD value of the strain reduced by OD_c_ value OD = average OD of a strain – OD_c_). The OD_c_ value was calculated for each microtiter plate independently.

Based on the results obtained, the strains were divided into the following categories: no biofilm producer (0 to 1 or more), weak biofilm producer (1 to 2 or more), moderate biofilm producer (2 to 4 or more), and strong biofilm producer (more than 4): for those that do not produce biofilm, the OD of the strain was below of the established cutoff point (OD ≤ OD_c_); for weak biofilm producers, the OD of the strain was between the cutoff value and the double of the corresponding OD_c_ value (OD_c_ < OD ≤ 2OD_c_); for moderate producers, the OD of the strain was between two and four times the cutoff value (2OD_c_ < OD ≤ 4OD_c_); and for strong producers: the OD of the strain was four times above of the cutoff value (4OD_c_ < OD).

### MIC, minimum bactericidal concentration, minimum biofilm inhibitory concentration, and minimum biofilm eradication concentration.

The seven antibiotics used in the study were potentially useful both for inpatient treatment and for outpatient maintenance of PJIs. The following antibiotics were used for the susceptibility testing: amikacin (Sigma-Aldrich) (128 to 0.125 μg/mL), ceftriaxone (Sigma-Aldrich) (32 to 0.03 μg/mL), ceftazidime (Sigma-Aldrich) (32 to 0.03 μg/mL), ciprofloxacin (BioChemika) (32 to 0.03 μg/mL), colistin (Sigma-Aldrich) (128 to 0.125 μg/mL), gentamicin sulfate (Sigma-Aldrich) (128 to 0.125 μg/mL), and meropenem (Sigma-Aldrich) (32 to 0.03 μg/mL). All of the antibiotics were prepared as stock solutions and stored at –80°C. The quality of antimicrobial susceptibility solutions were checked using E. coli (ATCC 25922), P. mirabilis (ATCC 29906), and P. aeruginosa (ATCC 27853).

MIC values were determined by the microtiter method following the guidelines of the European Committee on Antimicrobial Susceptibility Testing (EUCAST) (46). The procedure involves preparing 2-fold dilutions of each antimicrobial agents transferred to 96-well microtitration plate containing Mueller-Hinton medium. Then, each well was inoculated with a microbial inoculum prepared in the same medium after dilution of standardized microbial suspension adjusted to 0.5 on the McFarland scale (turbidity standard). All isolates were subcultured onto tryptic soy agar (TSA) (BD, Germany) for 18 to 24 h at 37°C prior to testing a microbial inoculum suspension. The plates were then incubated for 24 h. MIC was determined manually as the lowest concentration of antimicrobial agent that completely inhibits growth of the organism as detected by the unaided eye. In those cases in which breakpoints were lacking in the EUCAST table, Clinical and Laboratory Standards Institute (CLSI) guidelines were used (47). The minimum bactericidal concentration (MBC) was determined using the flash microbicide method previously described (48). The MBC was defined as the minimum concentration required to kill a certain bacterial concentration. Briefly, 10 μL of each well were mixed after 24 h of incubation with 190 μL of tryptic soy broth in a new 96-well plate, which was further incubated statically at 37°C and 5% CO_2_ for 24 h. The MBC endpoint was determined as the lowest concentration of antibiotic at which there was no visible growth.

Minimal biofilm inhibitory concentrations (MBIC) and minimal biofilm eradication concentrations were determined using the methodology previously described (49). For MBIC, biofilm formation on pegs from the Calgary device was induced by inoculating 200 μL of tryptic soy broth containing 10^6^ CFU/mL of bacteria/well in a 96-well flat-bottom plate (Thermo Fisher Scientific, MA, USA). The lid (Thermo Fisher Scientific) of the Calgary device was then placed, and the plate was incubated in turmoil at 37°C and 5% CO_2_ for 24 h. After incubation, the pegs from the lid were rinsed two times in wells containing 200 μL of saline. Afterwards, the lid was placed in a plate with different concentrations of antibiotics with a 2-fold dilution were added to Cation-Adjusted Muller-Hinton Broth (CAMHB) to a final volume of 200 μL/well and was incubated by static incubation at 37°C and 5% CO_2_ for at least 20 h. After incubation, the MBIC value was determined by the naked eye considering the first dilution in which no bacterial growth was observed. The MBEC is the minimum concentration required to kill a bacterial biofilm. For MBEC, the lid from the MBIC was rinsed two times in a plate with wells containing 200 μL of saline 0.9% NaCl, placed in a plate with 200 μL of tryptic soy broth, and incubated statically at 37°C and 5% CO_2_ for 24 h. After incubation, MBEC was determined by the naked eye considering the first dilution in which no bacterial growth was observed.

### Statistical analysis.

For the data calculation, we categorized in four groups the bacteria adherence, based on three standard deviation above the mean OD of each negative control (30, 50). The results are represented as the medians (quartiles 1 to 3) and percentages. A nonparametric test was calculated using the Kruskal-Wallis and unpaired Wilcoxon tests for multiple comparisons between each species. Moreover, to study the association and correlation between biofilm formation and antimicrobial susceptibility categories, the Wilcoxon test was used. A *P* value of <0.05 was considered significant. For data analysis, Stata version 15.1 was employed. Continuous variables expressed in median and percentiles (p50 and p90) were calculated using Excel.
